# ADTKD-*UMOD* in a girl with a *de novo* mutation: A case report

**DOI:** 10.3389/fmed.2022.1077655

**Published:** 2022-12-20

**Authors:** Meng-shi Li, Yang Li, Lei Jiang, Zhuo-ran Song, Xiao-juan Yu, Hui Wang, Ya-li Ren, Su-xia Wang, Xu-jie Zhou, Li Yang, Hong Zhang

**Affiliations:** ^1^Renal Division, Peking University First Hospital, Beijing, China; ^2^Kidney Genetics Center, Peking University Institute of Nephrology, Beijing, China; ^3^Key Laboratory of Renal Disease, Ministry of Health of China, Beijing, China; ^4^Key Laboratory of Chronic Kidney Disease Prevention and Treatment (Peking University), Ministry of Education, Beijing, China; ^5^Renal Pathological Center, Institute of Nephrology, Peking University, Beijing, China; ^6^Laboratory of Electron Microscopy, Pathological Centre, Peking University First Hospital, Beijing, China

**Keywords:** ADTKD, *UMOD*, *de novo* mutation, case report, genetic kidney disease

## Abstract

Autosomal dominant tubulointerstitial kidney disease due to *UMOD* mutations (ADTKD-*UMOD*) is a rare condition associated with high variability in the age of end-stage kidney disease (ESKD). An autosomal dominant inheritance is the general rule, but *de novo UMOD* mutations have been reported. It was reported that the median age of ESKD was 47 years (18–87 years) and men were at a much higher risk of progression to ESKD. Here, we reported a 13-year-old young girl with unexplained chronic kidney disease (CKD) (elevated serum creatine) and no positive family history. Non-specific clinical and histological manifestations and the absence of evidence for kidney disease of other etiology raised strong suspicion for ADTKD. Trio whole-exome sequencing confirmed that she carried a *de novo* heterozygous mutation c.280T > C (p.Cys94Arg) in the *UMOD* gene. The functional significance of the novel mutation was supported by a structural biology approach. With no targeted therapy, she was treated as CKD and followed up regularly. The case underscores the clinical importance of a gene-based unifying terminology help to identify under-recognized causes of CKD, and it demonstrates the value of whole-exome sequencing in unsolved CKD.

## 1 Introduction

ADTKD is a rare genetically heterogeneous disorder characterized by slowly progressive loss of kidney function, and bland urinary sediment with absent or trace proteinuria. A gene-based sub classification has been proposed by Kidney Disease: Improving Global Outcomes (KDIGO) (1). The most prominent features of Autosomal dominant tubulointerstitial kidney disease due to *UMOD* mutations (ADTKD-*UMOD*) include early onset hyperuricemia (gout) and/or a family history of hyperuricemia (2, 3). Compared to ADTKD-*MUC1*, ADTKD-*UMOD* seems to be associated with an earlier age at disease presentation but a later age to end-stage kidney disease (ESKD) (4). The age for development of ESKD ranges from 18 to 87 years, and renal fibrosis is the common feature (2). Because those clinical manifestations are mostly non-specific, so the diagnosis of ADTKD in practice is difficult and currently relies on positive family history and genetic sequencing (1, 2).

## 2 Case report

**FIGURE 1:**
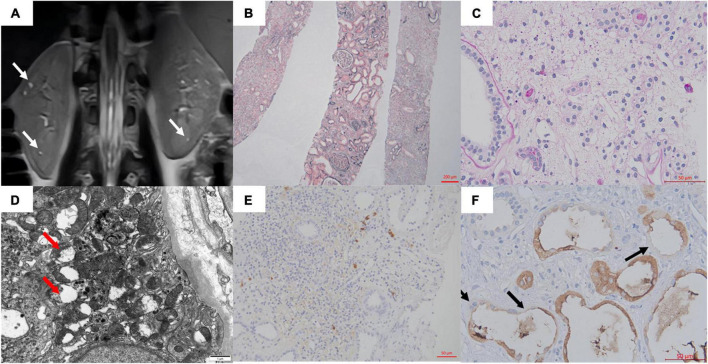
**(A)** MRI showed multiple microscopic cysts in the medulla of the patient’s kidney. **(B,C)** Interstitial edema and tubular destruction (PAS 400×). **(D)** Transmission electron micrograph of tubular epithelial cytoplasm showed mitochondrial swelling, focal cristae damage, and vacuolation (arrows). **(E)** The IgG4 staining in the interstitium. **(F)** Uromodulin was expressed in the cytoplasm of most cells of the thick ascending limb of Henle. Some epithelial cells were negative or weakly/partial UMOD positive along the luminal side (arrows). No UMOD protein accumulation was observed (400×).

A 13-year-old girl presented to our hospital with a 1-month history of abnormal renal function. She was a student of middle school and had no special medical, family, or psycho-social history. On a physical examination, she had a serum creatinine of 164 μmol/L (Schwartz Pediatric eGFR 40 ml/min), urea 15.1 mmol/L, and blood pressure 140/90 mmHg, without any discomfort. She had an elevated serum uric acid level of 438 μmol/L but hadn’t experienced gout symptoms. Urine sediment analysis was normal, and 24 h urine protein was only 0.24 g/2,000 ml. She had some clinical features of tubular injury, such as low morning urine osmolality (312 mOsm/kg), high level of urinary α1 microglobulin (22.9 mg/L, range 0–12 mg/L), and compromised renal acid-base handling ability, including lower urine bicarbonate (2.7 mmol/L, normal range < 26.8 mmol/L), titratable acid (4.3 mmol/L, normal range > 10.5 mmol/L), and ammonium ion (0.2 mmol/L, normal range > 25.2 mmol/L). Her hemoglobin level was normal (120 g/L) but iPTH (556.7 μg/ml, normal range 15–65 μg/ml) was elevated. Detailed clinical information was listed in Table 1. She had normal sized kidney of 10.5 cm in length. However, magnetic resonance imaging (MRI) of the kidneys revealed some occasional cysts at the corticomedullary boundary (Figure 1A). Without any indications of a thick-walled bladder, ureteric dilatation, and hydronephrosis for urological anomalies, the family refused voiding cystourethrography (VCUG).

**TABLE 1:** Clinical information and treatment of the patient.

Item	Value	Normal range
Hemoglobin	120 g/L	115–150 g/L
Albumin	38.5 g/L ↓	40–55 g/L
White blood cells	9*10^∧^9/L	3.5–9.5*10^∧^9/L
Serum creatinine	164 μmol/L ↑	44–133 μmol/L
Urea	15.1 mmol/L ↑	1.8–7.1 mmol/L
eGFR	40 ml/min ↓	
Uric acid	438 μmol/L ↑	90–360 μmol/L
Na^+^	137.79 mmol/L	135–145 mmol/L
K^+^	3.57 mmol/L	3.5–5.5 mmol/L
Urinary α1 microglobulin	22.9 mg/L ↑	0–12 mg/L
Urinary N-Acetyl-B -D-Glucosaminidase	6.5 U/L	0.3–12 U/L
Urine red blood cell	1.2/HP	0–7/HP
24 h urine protein	0.24 g/2,000 ml ↑	0–0.15 g/24 h
Urine pH	6.0	4.5–8.0
Urine bicarbonate	2.7 mmol/L	0–26.8 mmol/L
Titratable acid	4.3 mmol/L ↓	> 10.5 mmol/L
Ammonium ion	0.2 mmol/L ↓	> 25.2 mmol/L
Urine osmolality	312 mOsm/kg ↓	600–1,000 mOsm/kg
Treatment	**BP control:** Amlodipine 2.5 mg bid; Betaloc 47.5 mg qd	
	**Hyperuricemia control:** Febuxostat 20 mg qd; Sodium bicarbonate: 0.5 g tid	
	Prednisone 40 mg qd (Gradual tapering and discontinuation after 6 months)	

The immunoglobulin levels were normal and no autoantibodies were found. The patient’s IgG4 was mildly elevated at 2.48 g/L (normal range < 2.01 g/L), and C3 was slightly decreased at 0.59 g/L (normal range 0.6–1.5 g/L). MRI showed no evidence of other organ involvement. The patient denied any recent infections, toxic drug exposure, or any family history of kidney disease. Secondary hypertension due to obstructive sleep apnea, primary aldosteronism, pheochromocytoma, hyperthyroidism, renovascular disease, renal artery stenosis, aortic coarctation, and inflammatory or systemic conditions were also ruled out.

With informed consent, a kidney biopsy was performed to further identify the etiology and the severity of the tubulointerstitial injury. No evidence of immune complex deposition or inflammation or cell proliferation. Light microscopy showed tubulointerstitial fibrosis, atrophy, cystic dilatation of tubules, and widely distributed global glomerulosclerosis (83%). Electron microscopy showed mitochondrial swelling, focal cristae damage, and vacuolation of tubular cells (Figures 1B–D). Glomerulonephritis, IgG4-related disease, and alport syndrome were ruled out (Figure 1E).

**FIGURE 2:**
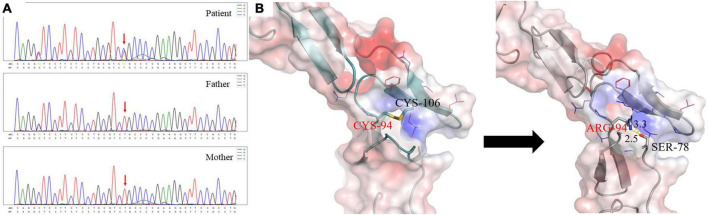
**(A)** The patient carried a heterozygous mutation in the UMOD gene c.280T > C (p.C94R), and neither of her parents carried this mutation. **(B)** Different interaction patterns in wild type and p.C94R mutant. The left one is for the wild-type structure and the right one is for the p.C94R mutant. The electrostatic surface is colored according to a scale from red (negative, min = −5.0 kT/e) to blue (positively, max = 5.0 kT/e). The backbone structure is shown in cartoon representation, Cys94, Cys106, and Ser78 as sticks with the rest of the side chains as lines. The distance between the hydrogen donor and the hydrogen acceptor is marked in the unit of Angstrom (Å).

According to the above results, she was suspected of a possible diagnosis of ADTKD. However, abnormal UMOD accumulations, typically as polymorphic unstructured materials by PAS staining, were not noteworthy (Figure 1F). Thus the trio whole-exome sequencing was then performed. A *de novo* heterozygous mutation c.280T > C (p.Cys94Arg) in the *UMOD* gene was found, which was absent in her parents (Figure 2A). The mutation was predicted to be deleterious and has been reported in a clinical case associated with hyperuricemic nephropathy (5). A different amino acid substitution at the same site (p.Cys94Trp) has been reported in patient with ADTKD (2). According to the 2015 ACMG genetic variant classification criteria and guideline (6), it was defined as a likely pathogenic variant (PS2 + PM5 + PP3). For molecular function, the mutation was located in the EGF-2 domain (position 65–107), which was associated with calcium binding. To further predict the effect of the mutation on the tertiary structure of the protein, we created a 3D structural model of UMOD protein using Phyre2 (7) and analyzed the mutated protein using Missense3D (8), VarSite (9), and PyMOL (10). Results showed that the substitution disrupted the disulfide bond with its interacting residue Cys106 (Distance: 2.151 Å) on the chain, triggering a clash alert, with the predicted local clash score for the mutant and the wild type being 87.17 and 40.69, respectively. In addition, the mutant arginine was more hydrophilic, which might prefer protein surface to the interior and result in protein misfolding (Figure 2B).

We gave the patient supportive treatment (1, 11) of amlodipine, betaloc for blood pressure control, and febuxostat, sodium bicarbonate for hyperuricemia. Because initially we cannot rule out immune-related kidney injury with her abnormal levels of C3 and IgG4, prednisone with an initial dose of 40 mg per day was tentatively added. However, it showed no beneficial effect and was stopped 6 months later. The detailed regimen was listed in Table 1. The patient has been followed up over 2 years till date. Her serum creatinine gradually elevated to about 345 μmol/L (Schwartz Pediatric eGFR 16 ml/min), despite her well-controlled serum uric acid (< 320 μmol/L), urine protein (< 0.5 g/24 h), and blood pressure (around 120/80 mmHg).

## 3 Discussion

We presented the case of a 13-year-old girl with unexplained abnormal renal function with the main manifestation of renal tubular interstitial damage. Despite her reported negative family history, the patient was suspected of genetic kidney disorder after a careful workshop of both clinical and pathological investigations. Using the new clinical *UMOD*-scoring system proposed in 2020 (2), our patient had a score of 6 (over 5 was suggested as a possibility of ADTKD-*UMOD*). Therefore, trio WES was performed and a *de novo* mutation in the *UMOD* gene was identified. According to the current KDIGO diagnostic criteria, positive family history was a prerequisite in establishing the diagnosis of ADTKD (1). But a negative family history may not exclude the diagnosis. There have been a few case reports with *de novo* mutations in *UMOD* (4). We highlighted that, for some young patients (positive or negative family history) with unexplained decreased kidney function, the possibility of hereditary kidney disease needs to be considered and genetic sequencing needs to be actively performed.

In addition to genetic diagnosis, in this case, MRI of the kidneys revealed some occasional cysts at the corticomedullary boundary, which was characteristic of ADTKD. And the effectiveness of MRI for the identification of ADTKD cysts has been confirmed in previous reports (12). Besides, immunohistochemistry may provide clues such as abnormalities in uromodulin staining, i.e., coarsely granular cytoplasmic staining or perinuclear positivity in flattened tubular epithelial cells in the loop of Henle epithelium. But we did not observe this feature in our case.

Due to the lack of large-scale epidemiological studies of ADTKD-*UMOD*, the proportion of patients with early onset chronic kidney disease (CKD) or ESRD was still unclear. However, cases have been reported of the onset of ADTKD-*UMOD* in adolescents and even in children (13). *De novo* mutations resulting in ADTKD-UMOD are rare. Previously, many of these families were undiagnosed and uncertain of the cause of inherited kidney disease. Patients with ADTKD-UMOD develop slowly progressive CKD, with a median age of ESKD of 47 years (14). But kidney disease progression is highly variable between and within families. Some individuals may develop ESKD in their 20 s, while others may not require kidney replacement therapy until past 70 years of age (14). As for correlation between genotype and phenotype, a previous study showed that patients with mutations in the EGF domain was at a higher risk of early onset ESRD (15). The early onset of CKD and ESRD may be related to the impaired global protein structure caused by mutations in EGF domain (15). This needs to be confirmed by further cases and experiments in the future.

The known genes causing ADTKD include *UMOD*, *MUC1*, *HNF1B*, *REN*, and *SEC61A1*. And mutations in *UMOD* are the most common type found in up to 3% of monogenic CKD patients (16). *UMOD* gene is located at 16p12.3, and it encodes the uromodulin protein, which is expressed exclusively by epithelial cells of the thick ascending limb of Henle’s loop (TALH) and distal convoluted tubule lumen (17). By 2021, a total of 135 *UMOD* mutations have been reported, most of which are located within the 30–300 sites near the N terminus containing the EGF-1, EGF-2, and D8C domains, and 53.8% of all mutations were cysteine substitutions (2). The mutation in our current case is located in the hotspot and is also a cysteine substitution. It has been reported that mutations of disulfide bonds in UMOD can lead to partial endoplasmic reticulum (ER) retention and then trigger ER stress with unfolded protein response, which eventually leads to activation of proinflammatory signals and cell death (18–20).

ADTKD currently lacks treatment options and has a poor prognosis, requiring international cooperation and rare disease organizations to establish a disease registry. Considering the gain-of-toxic-function effect of mutant UMOD accumulated in the ER, decreasing the amount of mutant uromodulin production might be a potential treatment strategy. In the future, gene editing and stem cell research might be explored.

In conclusion, the current case emphasized some lessons for nephrologists to be learned in the era of genetics. This should enhance recognition and correct diagnosis of affected individuals, facilitate genetic counseling, and stimulate research into the underlying pathophysiology.

## Ethics statement

This study protocol was reviewed and approved by the Biomedical Research Ethics Committee, Peking University First Hospital, approval number (2018-099). Written informed consent from the patients/participants OR patients/participants legal guardian/next of kin was not required to participate in this study in accordance with the national legislation and the institutional requirements. Written informed consent was obtained from the patient’s parents for publication of this case report and any accompanying images.

## Author contributions

M-SL, YL, and Z-RS collected the data, conceived, and wrote the manuscript. LJ, X-JY, HW, Y-LR, and S-XW provided with the figures. X-JZ revised the manuscript critically for important intellectual content and supervised the research group and gave the final approval of the version to be published. HZ and LY edited the manuscript and gave the final approval of the version to be published. All authors contributed to the article and approved the submitted version.
